# Antiviral efficacy of LNP-delivered IFN-α14-ApoAI mRNA for chronic hepatitis B

**DOI:** 10.1016/j.jve.2026.100627

**Published:** 2026-06-03

**Authors:** Qunling Yang, Qiang Li, Jie Cao, Conglin Zhao, Mengxin Lu, Shuangshuang Sun, Mingsheng Chen, Chong Chen, Yuxian Huang, Shuai Tao, Liang Chen

**Affiliations:** a The Diagnosis and Treatment Center of Liver Disease, Shanghai Public Health Clinical Center, Fudan University, No. 2901 Caolang Road, Jinshan District, Shanghai, China; b Department of Infectious Diseases, Shanghai Public Health Clinical Center, Fudan University, Shanghai, China; c Department of Basic Research, Shanghai Public Health Clinical Center, Fudan University, Shanghai, China

**Keywords:** Hepatitis B virus, IFN-α14, mRNA therapy, Lipid nanoparticles, Pegylated interferon

## Abstract

Chronic hepatitis B virus (HBV) infection remains a major global health burden, and current therapeutic options such as pegylated interferon-α (PEG-IFNα) yield limited clinical efficacy. Here, we developed a lipid nanoparticle (LNP) formulation encapsulating mRNA encoding an IFN-α14-ApoAI fusion protein and evaluated its anti-HBV activity and safety profile in humanized IFNAR mouse models.

First, adeno-associated virus (AAV)-mediated delivery of IFN-α14-ApoAI provided preliminary evidence of safe and sustained HBV suppression in mice, suggesting the feasibility of gene delivery of this fusion protein for anti-HBV therapy. On this basis, we formulated IFN-α14-ApoAI mRNA into SM-102-based LNPs, designated as IFN-α14 LNP. A single intravenous injection of this LNP formulation showed a trend toward dose-dependent reduction of HBV antigens. Furthermore, a single intravenous dose of 4 μg IFN-α14 LNP showed comparable inhibition of HBV antigens to the clinically approved drug PEG-IFNα2 (2 μg, subcutaneously). Moreover, safety assessment showed that the 4 μg dose was well tolerated with no detectable organ toxicity, only transient and self-limited cytokine elevations, and reversible splenic immune activation. In addition, repeated dosing of IFN-α14 LNP in mice induced neutralizing antibodies against the xenogeneic human IFN-α14, a limitation that is not anticipated in humans due to immune tolerance to self-proteins.

Collectively, our findings demonstrate that IFN-α14 LNP exerts anti-HBV activity while exhibiting a favorable safety profile in humanized IFNAR mouse models. It represents a novel therapeutic candidate for chronic hepatitis B that still requires refinement.

## 1 Introduction

The World Health Organization (WHO) estimates that approximately 254 million individuals worldwide are living with chronic hepatitis B virus (HBV) infection, with nearly 1.2 million annual fatalities attributable to HBV-induced liver cirrhosis and hepatocellular carcinoma.^1^

Current therapeutic regimens for chronic hepatitis B (CHB) comprise pegylated interferon (PEG-IFNα) and nucleos(t)ide analogues (NUCs). NUCs specifically target the viral reverse transcriptase activity, yet demonstrate limited efficacy in suppressing viral protein expression. Consequently, attainment of sustained virological response remains challenging, necessitating indefinite administration in most cases.^2^ In contrast, interferon therapy exerts multifaceted antiviral and immunomodulatory effects through diverse signaling pathways. Notwithstanding its adverse effect profile and parenteral administration requirements, interferon therapy offers distinct advantages including a defined treatment duration, absence of drug resistance, elevated rates of hepatitis B e antigen (HBeAg) seroconversion, and the potential for hepatitis B surface antigen (HBsAg) seroclearance.^3^ Clinical data demonstrate HBsAg clearance rates of 3-7% after 48 weeks of PEG-IFNα therapy, escalating to 11% after three years of treatment cessation - outcomes unattainable with NUC monotherapy.^4^ However, the constrained response rates and suboptimal tolerability observed in the majority of CHB patients substantially limit the clinical utility of interferon-based regimens, thereby driving investigative efforts toward optimization of interferon therapeutics and development of novel interferon formulations.

The development of novel human interferon-α subtypes represents a promising strategy for enhancing therapeutic efficacy in CHB management. The human IFN-α family encompasses 13 distinct subtypes, and accumulating evidence indicates subtype-specific variations in antiviral potency.^5^ Notably, subtype 14 (IFN-α14) has been identified as the most potent inhibitor of HBV covalently closed circular DNA (cccDNA) transcription and viral antigen production among all IFN-α subtypes in both in vitro and in vivo models. Mechanistically, IFN-α14 binds IFNAR1 with high affinity and uniquely elicits a concerted activation of both IFN-α and IFN-γ signaling pathways, leading to robust induction of a broad repertoire of ISGs.^6^

In contrast to plasmid DNA and viral vectors, mRNA delivery offers distinct advantages, as it requires only cytoplasmic entry to transiently initiate protein translation, eliminates the risk of insertional mutagenesis, and circumvents side effects such as viral vector-associated immune-mediated hepatitis. Nevertheless, mRNA faces significant challenges, including susceptibility to RNase degradation and poor permeability across cellular membranes.^7^ Lipid nanoparticles (LNPs) protect mRNA from nuclease degradation, facilitate cellular uptake and cytosolic release, and have emerged as the leading delivery platform for mRNA therapeutics. Following intravenous administration, LNPs accumulate preferentially in the liver through binding to apolipoprotein E (ApoE) and subsequent uptake via ApoE receptors on hepatocytes.^8^ This inherent hepatocyte-targeting property positions mRNA-LNPs as a promising platform for the treatment of liver diseases and liver-directed therapeutic interventions. Ionizable cationic lipids, including ALC-0315 and SM-102, have been utilized in SARS-CoV-2 mRNA vaccine formulations and have demonstrated a favorable safety profile.^9^ The LNP-based mRNA delivery platform has been widely employed in protein replacement strategies for a range of diseases.^10^

In the present study, we sought to enhance the efficacy of interferon-based therapy by harnessing the potent antiviral activity of IFN-α14 and the LNP-based mRNA delivery platform to develop a novel therapeutic intervention for HBV infection. Based on reports that fusion to apolipoprotein A-I (ApoAI) has been shown to mitigate the hematological toxicity of IFNα while enhancing its liver-targeting and immunostimulatory properties,^11^^,^^12^ we designed an IFN-α14-ApoAI fusion construct. After confirming its anti-HBV efficacy and safety via AAV-mediated delivery, we formulated mRNA encoding this fusion protein into SM-102-based LNP. In this study, we evaluated the anti-HBV activity of the LNP-formulated IFN-α14-ApoAI mRNA in a humanized IFNAR mouse model with persistent HBV replication. We compared the antiviral efficacy of a single intravenous dose of IFN-α14 LNP (4 μg) with the clinically approved drug PEG-IFNα2 (2 μg, subcutaneously). Additionally, we conducted a comprehensive safety assessment of IFN-α14 LNP, including body weight, serum chemistry, cytokine responses, and histopathology of major organs. To our knowledge, this work represents the first report of an LNP-formulated interferon-α mRNA therapeutic, establishing a novel and promising approach for the treatment of chronic hepatitis B.

## 2 Materials and methods

### 2.1 AAV vectors

AAV vectors were constructed using adeno-associated virus serotype 8 (AAV8). The hepatocyte-specific thyroxine-binding globulin (TBG) promoter was used to drive transgene expression. Human IFN-α14 or the fusion protein ORF (open reading frame), generated by linking human IFN-α14 (GenBank Accession Number: NM_002172.3) to mouse ApoA-I (NM_009692.4), were inserted into the AAV backbone, resulting in AAV8-TBG-IFN-α14 and AAV8-TBG-IFN-α14-ApoAI. A control vector expressing red fluorescent protein (AAV-RFP) was also constructed. Viral packaging, purification, and quantification were performed as described previously by Wu et al.^13^ Viral titers are expressed as viral genomes per milliliter (vg/mL).

### 2.2 mRNA synthesis and LNP preparation

The coding sequence of fusion protein IFN-α14-ApoA-I or control EGFP was chemically synthesized and cloned into the pCZ transcription plasmid (Genewiz Biotechnology Co., Ltd, Suzhou, China) under the control of the T7 promoter. The construct was flanked by the 5′ and 3’ untranslated regions (UTRs) of the human β-globin gene and included a 120-nt polyA tail. The resulting plasmid was linearized with *Bsp*QI and used as template for in vitro transcription with an mRNA synthesis kit (Genewiz Biotechnology Co., Ltd, Suzhou, China). After transcription, the template DNA was removed by DNase I treatment. The synthesized mRNA was purified by lithium chloride precipitation and dissolved in 25 mM sodium acetate (pH 5.2).

Lipid mixtures were formulated by Genewiz Biotechnology Co., Ltd (Suzhou, China) by dissolving the following components in ethanol at a molar ratio of 50:38.5:10:1.5 for cationic lipid SM-102, 1,2-distearoyl-sn-glycero-3-phosphocholine (DSPC), cholesterol, and 1,2-dimyristoyl-rac-glycero-3-methoxypolyethylene glycol-2000 (DMG-PEG2000). mRNA was prepared in 100 mM citric acid buffer (pH 4.0). The SM102 LNP and mRNA solutions were combined at a 1:3 flow ratio using an INano L+ microfluidic mixer (Micro&Nano Biologics, Shanghai, China) at a total flow rate of 12 mL/min. The resulting IFN-α14 LNP or EGFP LNP particles were diluted in PBS (pH 7.4), concentrated via centrifugal filtration (Amicon, 100 kDa, 3000g, 15min). Particle size was measured with a Zetasizer Nano ZS (Malvern Panalytical Ltd, Malvern, UK), and mRNA encapsulation efficiency was quantified using the Quant-iT™ RiboGreen RNA Assay Kit (Invitrogen, Carlsbad, CA, USA).

### 2.3 Animal experiments

All animal experiments were performed in accordance with ethical guidelines for animal research and the ARRIVE guidelines. All procedures were approved by the Shanghai Public Health Clinical Center Laboratory Animal Welfare & Ethics Committee. Only male animals were used in this study.

IFNAR-hEC mice (C57BL/6N mouse strain)^14^ carry a knock-in of the human IFNAR extracellular domain. Heterozygous mice were used for in vivo efficacy studies. For AAV treatment, mice (aged 6-8 weeks) were subjected to hydrodynamic injection (HDI) of the BPS replicon.^15^ After stable HBV viremia was confirmed at 28 days post-HDI, mice received intravenous injection of 1 × 10^11^ vg/mouse of AAV-IFN-α14, AAV-IFN-α14-ApoAI, or AAV-RFP (control).

For IFN-α14 LNP and PEG-IFN-α2b treatment, mice (aged 6-8 weeks) were transduced with 1 × 10^10^ vg/mouse of rAAV-HBV1.3 (genotype D, serotype ayw; Fubio, Suzhou, China) via tail vein injection to establish persistent HBV replication.^16^ After stable HBV viremia was confirmed at 28 days post-transduction, Mice were stratified by baseline serum HBsAg levels and then assigned to treatment groups to ensure comparable distribution of baseline antigen levels across groups. IFN-α14 LNP or control LNP (either empty LNP with identical lipid composition, or EGFP LNP, as specified in the respective figure legends) were administered intravenously (i.v.), while PEG-IFN-α2b (Amoytop, Xiamen, China) was injected subcutaneously (s.c.).

### 2.4 Enzyme-linked immunosorbent assay (ELISA)

Serum levels of HBsAg and HBeAg were determined using ELISA kits according to the manufacturer's instructions (KHB, Shanghai, China). Concentrations of IFN-α14 and IFN-α2 in serum were measured using a Human IFN-alpha multi-subtype ELISA kit (Catalog No. 41110; PBL, USA).

### 2.5 Statistical analysis

All data were analyzed using GraphPad Prism version 8.0 (GraphPad Software, San Diego, CA, USA). Two-way repeated-measures ANOVA with Tukey's (Fig. 4B-4C) or Bonferroni's (Fig. 5A-5D) post-hoc test was used for repeated-measures data. Unpaired *t*-test was used for Supplementary Fig. S1, and Kruskal-Wallis test with Dunn's post-hoc test for Supplementary Figure S5 A p-value <0.05 was considered statistically significant.

## 3 Results

### 3.1 Conceptual validation of IFNα14 gene therapy using the AAV8-TBG-IFNα14-ApoAI vector

**Fig. 1:**
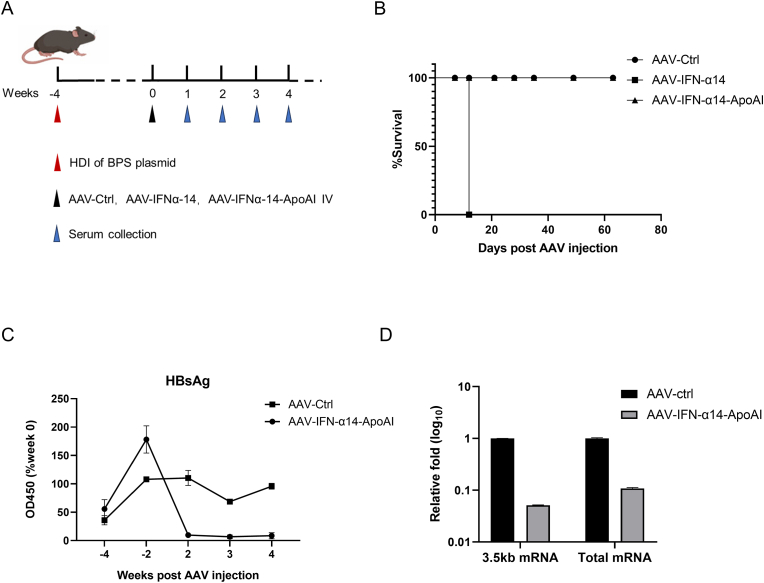
**Trend toward HBV transcription inhibition and survival benefit of AAV-delivered IFN-α14-ApoAI.** (A) Schematic diagram of AAV injection in the BPS-based HBV persistence mouse model established in IFNAR-hEC mice. (B) Survival curve of mice after AAV injection (n = 6). (C) HBsAg levels in mouse serum after AAV injection (n = 2 for AAV-Ctrl group, n = 3 for AAV-IFN-α14-ApoAI group). (D) HBV RNA levels in mouse liver tissues detected by qPCR at 4 weeks post-AAV injection (n = 1). No statistical comparisons were performed for panels C and D (pilot study, n = 1-3).

We first evaluated the therapeutic potential of IFN-α14 gene therapy for chronic hepatitis B. To this end, we constructed AAV8 vectors encoding human IFN-α14 under the control of a hepatocyte-specific TBG promoter, either alone (AAV-IFN-α14) or fused to murine ApoA-I (AAV-IFN-α14-ApoAI). ApoA-I fusion has been proved to enhance liver targeting and improve the safety of interferon-based therapies.^11^^,^^12^ IFNAR-humanized mice^14^ with persistent HBV replication, established via hydrodynamic injection of the BPS replicon,^15^ were treated with the two AAVs or a control AAV (as illustrated in Fig. 1A). Treatment with AAV-IFN-α14-ApoAI enabled survival over 60 days, Whereas AAV-IFN-α14 alone resulted in lethal toxicity within two weeks (Fig. 1B). Notably, both constructs showed a trend of reduction in serum HBsAg levels within two weeks (Fig. 1C and Supplementary Fig. S1). These observations are consistent with previous reports showing that fusion with ApoA-I can assure safety while retaining the efficacy of IFN-α delivered by AAV.^11^^,^^12^ Moreover, AAV-IFN-α14-ApoAI showed a trend toward suppression of intrahepatic 3.5 kb and total HBV RNA to approximately 10% of control levels (Fig. 1D), without reducing BPS plasmid DNA (Supplementary Fig. S2), providing preliminary evidence of direct transcriptional inhibition.

### 3.2 Preparation and characterization of IFN-α14-APOAI LNP

**Fig. 2:**
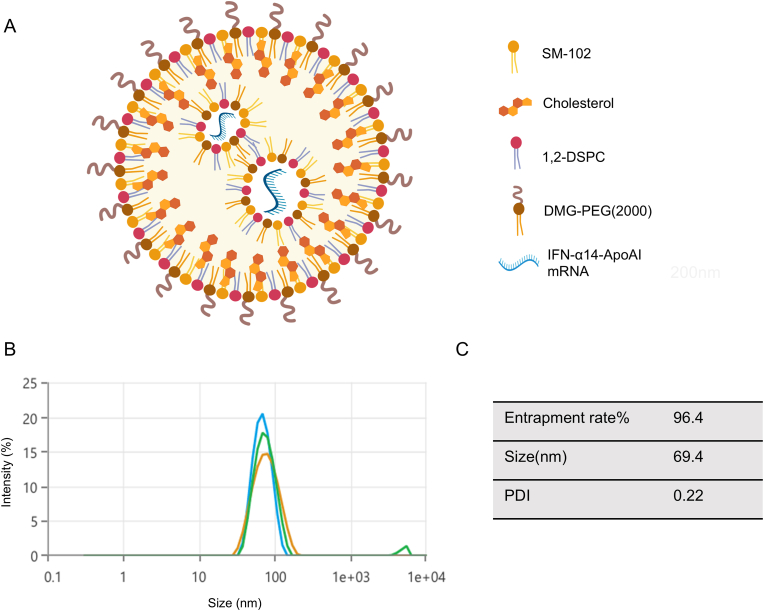
**Preparation and characterization of IFN-α14 LNP.** (A) Schematic representation of IFN-α14 LNP (Created in BioRender. Tao, S. (2026) https://BioRender.com/i0qg2kg). This is a conceptual illustration depicting the four lipid components (SM-102, DSPC, cholesterol, and DMG-PEG2000) and the encapsulation of mRNA. (B) The size of three IFN-α14 LNP samples was measured using dynamic light scattering. (C) Encapsulation efficiency and colloidal properties of IFNα14 LNP.

Based on these findings, we generated LNPs encapsulating IFN-α14-ApoAI fusion mRNA (hereafter termed IFN-α14 LNP for simplicity) using SM-102 and helper lipids (Fig. 2A). The LNPs were also characterized based on particle size, polydispersity index (PDI), and mRNA encapsulation efficiency. The results demonstrated that IFN-α14 LNP exhibited an average size of 69.4 nm (Fig. 2B and C), a high encapsulation efficiency of 96.4% and good homogeneity with a PDI of 0.22 (Fig. 2C), suggesting that the particles can efficiently extravasate from blood vessels and facilitate effective cellular uptake and internalization.

### 3.3 Dose-finding and antiviral activity of IFN-α14 LNP in vivo

**Fig. 3:**
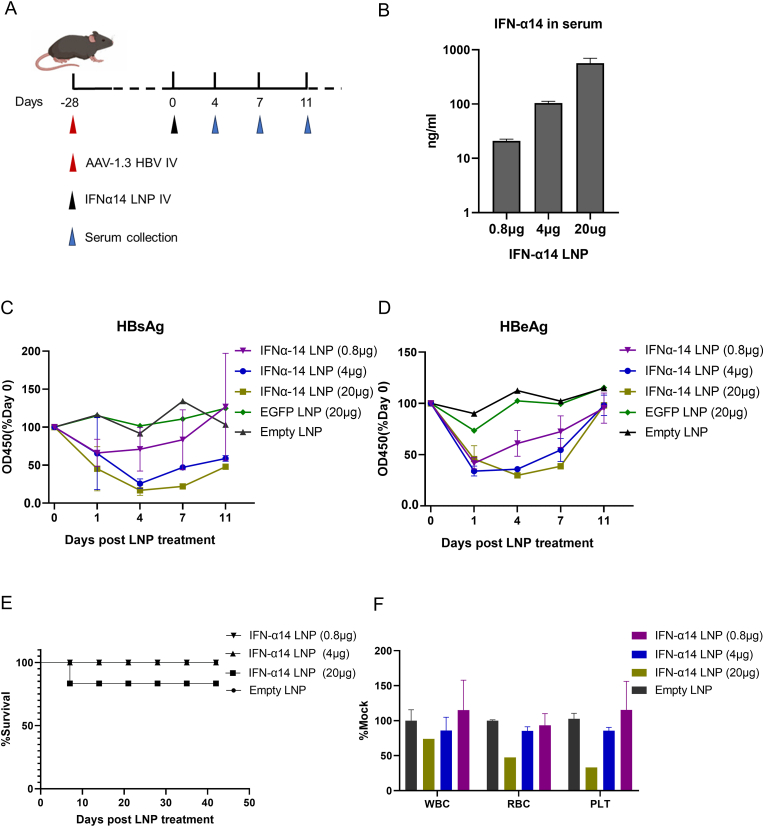
**Exploratory dose-finding and antiviral activity of IFN-α14 LNP in AAV-HBV-transduced IFNAR-hEC mice.** (A) Schematic diagram of LNP treatment in the AAV-HBV persistence mouse model established in IFNAR-hEC mice. (B) Serum IFN-α14 levels in mice at one day post-IFN-α14 LNP injection. n = 2 per group. Later time point data for the 4 μg dose are presented in Fig. 4D. (C) (D) Serum HBsAg and HBeAg levels following IFN-α14 LNP treatment. For 0.8 μg and 4 μg doses: n = 2. For 20 μg dose: n = 2 for days 0–4; n = 1 for days 7–11 (one mouse died on day 7). Control groups: EGFP LNP (20 μg mRNA, n = 1, high-dose irrelevant LNP); Empty LNP (lipid content matched to the 4 μg IFN-α14 LNP, n = 1). Data were normalized to each mouse's own baseline (Day 0 = 100%). (E) Survival curve of mice after IFN-α14 LNP treatment. n = 6 per group. (F) Measurement of white blood cell (WBC), red blood cell (RBC), and platelet (PLT) counts (% of mock) in mice at 7 days post IFN-α14 LNP treatment. n = 2 for 4 μg, 0.8 μg, and Empty LNP groups; n = 1 for 20 μg (exploratory). For groups with n = 2, data are shown as mean ± SD. For groups with n = 1, only the individual value is shown without an error bar. Due to the exploratory, dose-finding nature, no statistical comparisons were performed.

To evaluate the in vivo antiviral efficacy of IFN-α14 LNP, we established an AAV-HBV infection model in IFNAR-hEC transgenic mice. After four weeks, upon stabilization of serum HBV antigens, the mice received a single intravenous injection of IFN-α14 LNP at five-fold serial doses (20, 4, and 0.8 μg of encapsulated mRNA) and control LNPs (EGFP LNP, n = 1; empty LNP, n = 1) (Fig. 3A). A trend toward dose-dependent elevation of serum IFN-α14 levels was observed in mice one day post-injection, spanning from 10 to 1000 ng/ml (Fig. 3B). Serum S and E antigen levels were monitored on days 1, 4, 7, and 11 post-treatment. For each dose, two mice were evaluated and one death occurred in the 20 μg group on day 7 (n = 2 initially, n = 1 thereafter). IFN-α14 LNP treatment showed a trend toward dose-dependent antiviral activity, in contrast to the control LNP (Fig. 3C & D). Individual mouse data of HBsAg are provided in the Supplementary Fig. S3.

**Fig. 4:**
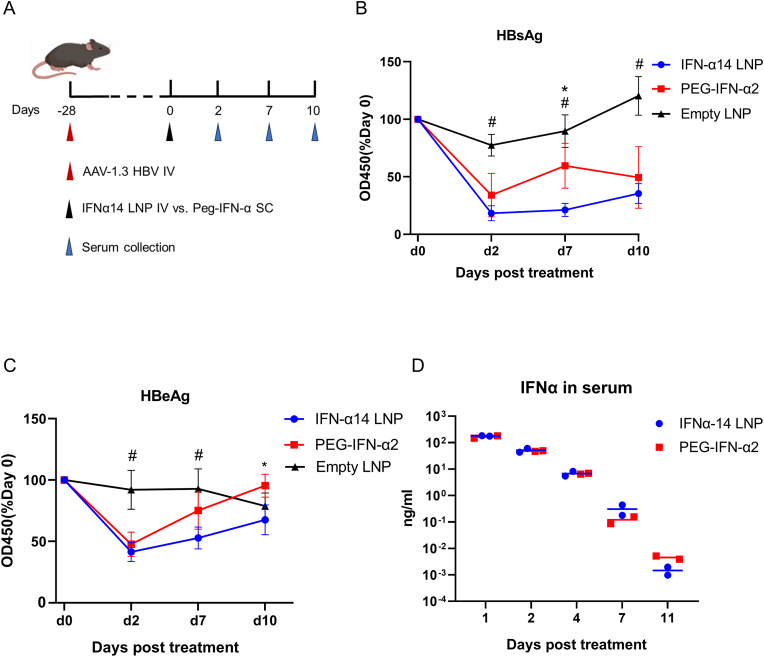
**Antiviral activity of IFN-α14 LNP and PEG-IFN-α2 in AAV-HBV-transduced IFNAR-hEC mice.** (A) Schematic diagram of the treatment. AAV-HBV-transduced IFNAR-hEC mice received a single intravenous injection of empty LNP (lipid composition matched to 4 μg IFN-α14 LNP), IFN-α14 LNP (4 μg), or a single subcutaneous injection of PEG-IFNα2 (2 μg). (B) Serum HBsAg and (C) HBeAg levels at days 0, 2, 7, and 10 post-treatment. Data are shown as mean ± SD (n = 5 per group). ∗P < 0.05 (IFN-α14 LNP vs. PEG-IFNα2), #P < 0.05 (IFN-α14 LNP vs. empty LNP). (D) Serum IFN-α levels in the treatment groups (n = 2 per group). Data are shown as individual values with the mean indicated by a short horizontal line.

**Fig. 5:**
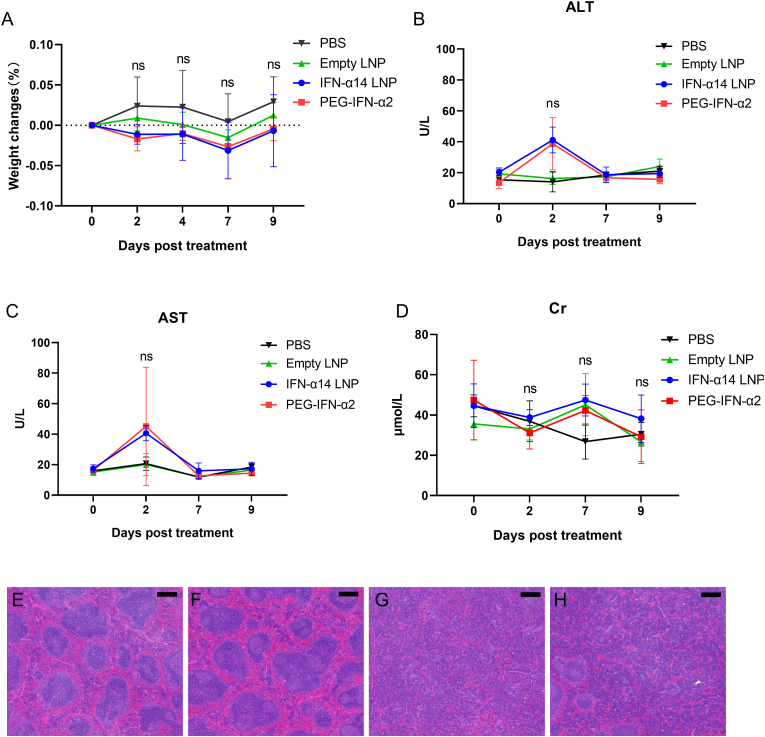
**Safety and pharmacodynamic assessment of IFN-α14 LNP.** AAV-HBV-transduced IFNAR-hEC mice (n = 3 per group) received a single intravenous injection of mock (PBS), empty LNP (lipid composition matched to 4 μg IFN-α14 LNP), IFN-α14 LNP (4 μg), or a single subcutaneous injection of PEG-IFNα2 (2 μg). Blood was collected at the indicated time points (0, 2, 7, 9 days). Spleens and other major organs were harvested at day 9 for histopathology. (A) Body weight changes. (B-D) Serum ALT (B), AST (C), and creatinine (D) levels at days 0, 2, 7, and 9 post treatment. (E–H) Representative H&E-stained sections of spleens from mock (E), Empty LNP (F), IFN-α14 LNP (G), and PEG-IFNα2 (H) groups (40x; scale bar = 250 μm). Images are representative of n = 3 mice per group. Data in (A-D) are mean ± SD (n = 3 per group) with no statistically significant differences between any of the groups (mock, empty LNP, IFN-α14 LNP, and PEG-IFN-α2) at all timepoints.

In survival experiment, one death occurred in the high-dose (20 μg/mouse) group on day 7, leading to a survival rate of 83.3% in that group; all other groups exhibited 100% survival (Fig. 3E). In the 20 μg group (n = 1, exploratory), we observed a reduction in red blood cells and platelets, suggesting potential hematological toxicity at this dose (Fig. 3F).

### 3.4 Antiviral activity of IFN-α14 LNP and PEG-IFNα2 in vivo

Next, we compared the efficacy of IFN-α14 LNP with the current anti-HBV drug PEG-IFN-α2. Mice received a single subcutaneous injection of PEG-IFN-α2 at 2 μg per mouse, a regimen previously described by Wang et al.^17^ Based on this and the results in Fig. 3, we selected the dose of 4 μg/mouse of IFN-α14 LNP for further comparative study with PEG-IFN-α2 (as illustrated in Fig. 4A).

In AAV-HBV-transduced IFNAR-hEC transgenic mice, 4 μg IFN-α14 LNP (i.v.) and 2 μg PEG-IFN-α2 (s.c.) showed comparable suppression of HBsAg and HBeAg at day 2 (Fig. 4B and C). Notably, IFN-α14 LNP achieved a more sustained effect with slower HBsAg rebound. By day 7, IFN-α14 LNP was superior to PEG-IFNα2 in suppressing HBsAg, and maintained the suppression through day 10 (Fig. 4B). Serum IFNα levels were assessed at 1, 2, 4, 7 and 11 days post-injection. 4 μg IFN-α14 LNP and 2 μg PEG-IFN-α2 induced comparable serum IFNα levels (∼100 ng/mL) at day 1, with the two groups showing the same order of magnitude at the subsequent time points (Fig. 4D).

To explore potential molecular differences, we analyzed liver transcriptomes after 4 μg IFN-α14 LNP (i.v.) or 2 μg PEG-IFN-α2 (s.c.) treatment (Supplementary Fig. S4). Among differentially expressed genes, 422 were shared between the two treatments, while more were unique to IFN-α14 LNP (495) than to PEG-IFN-α2 (195) (Supplementary Fig. S4A). Focusing on differentially expressed ISGs, 25 out of 27 identified ISGs showed higher expression in the IFN-α14 LNP group compared to the PEG-IFN-α2 group, including anti-HBV effectors Mx1,^18^ Mx2,^19^ and IFIT family members^20^ (Supplementary Fig. S4B). Gene Ontology (GO) analysis confirmed enrichment in antiviral and innate immunity pathways (Supplementary Fig. S4C). These data indicate that 4 μg IFN-α14 LNP (i.v.) is associated with a broader ISG response than 2 μg PEG-IFN-α2 (s.c.), which correlates with its more sustained anti-HBV efficacy.

### 3.5 Safety and pharmacodynamic assessment of IFN-α14 LNP

To evaluate the safety profile of the IFN-α14 LNP formulation (4 μg, i.v.), we performed an independent study assessing body weight, serum chemistry, cytokine responses, and histopathology of major organs in AAV-HBV mouse model.

As shown in Fig. 5A, no significant differences in body weight were observed among the mock, empty LNP, IFN-α14 LNP (4 μg, i.v.), and PEG-IFNα2 (2 μg, s.c.) groups over the 9-day observation period. Similar to PEG-IFNα2, IFN-α14 LNP induced a mild, transient elevation of serum ALT and AST at day 2 (not statistically significant), which returned to baseline by day 7. This elevation was not observed in the empty LNP group (Fig. 5B and C), indicating it was attributable to the expressed interferon rather than the LNP vehicle. Creatinine levels remained normal in all groups (Fig. 5D), indicating no detectable hepatotoxicity or nephrotoxicity.

To assess acute inflammatory responses, we measured serum IL-6 and TNF-α at 3, 6, and 17 h post-treatment. The IFN-α14 LNP group showed a transient elevation of both cytokines at 3 h, which rapidly declined and returned to near baseline by 17 h, demonstrating a self-limited acute-phase response (Supplementary Fig. S5A and S5B). The empty LNP group showed no significant elevation, indicating a minimal acute cytokine response to the LNP vehicle itself.

During necropsy, spleens from the IFN-α14 LNP and PEG-IFNα2 groups were visibly enlarged and darkened (Supplementary Fig. S6). Histopathologically, both treatments induced marked white pulp expansion, fusion of lymphoid follicles, and blurring of the white pulp-red pulp boundary (Fig. 5E ∼ H), with the expanded areas composed predominantly of lymphocytes (Supplementary Fig. S7). These changes are consistent with the expected pharmacodynamic effect of IFN-α-mediated lymphocyte activation^21^^,^^22^ and were reversible, as splenic architecture was normal in mice examined four months post-dose (Supplementary Fig. S8). No significant histopathological changes were observed in the heart, liver, kidney, or lung in any treatment group (Supplementary Fig. S7).

Collectively, these data demonstrate that the 4 μg dose of IFN-α14 LNP is well-tolerated and does not induce detectable organ toxicity.

### 3.6 Immunogenicity of repeated IFN-α14 LNP administration

Since human IFN-α14 delivered by ionizable cationic lipids is xenogeneic to mice, repeated dosing may elicit antibodies and abrogate antiviral efficacy. To test this, we administered four consecutive doses. As shown in Supplementary Fig. S9A, IFN-α14 LNP failed to suppress HBsAg after the third dose, whereas PEG-IFN-α2 maintained its efficacy. Consistent with this, anti-human IFN-α14 antibodies were detected in mouse sera after the third administration (Supplementary Fig. S9B), indicating that the loss of antiviral activity is attributable to antibody generation against the human IFNα14.

## 4 Discussion

In this study, we developed an LNP-formulated IFN-α14-ApoAI mRNA therapeutic and evaluated its anti-HBV efficacy and safety profile in humanized IFNAR mice with persistent HBV replication. Our results provide exploratory evidence of dose -dependent antiviral activity of this LNP formulation. Under the specific conditions tested (4 μg i.v. vs. 2 μg s.c.), a single intravenous dose of IFN-α14 LNP showed comparable inhibition of HBV antigen to the clinically approved PEG-IFNα2 regimen. Comprehensive safety assessments further indicated that the 4 μg dose was well tolerated, with a favorable safety profile.

The AAV experiment showed that IFN-α14 gene transfer is feasible and that ApoAI fusion improves safety. AAV-IFN-α14 caused rapid lethality, whereas AAV-IFN-α14-ApoAI enabled survival beyond 60 days (Fig. 1B) and showed a trend toward reduction of HBsAg (Fig. 1C) and HBV RNA (Fig. 1D). The toxicity likely reflects extrahepatic hematological effects, as previously reported.^11^^,^^12^ Based on this validation, we formulated IFN-α14-ApoAI mRNA into LNPs (IFN-α14 LNP).

In dose-finding experiment, IFN-α14 LNP exhibited a dose-dependent trend in reducing HBsAg and HBeAg (Fig. 3C and D) and the 20 μg dose was associated with mortality and cytopenia (Fig. 3E and F). Based on these exploratory observations, we selected the 4 μg dose for further evaluation in Fig. 4, Fig. 5. As shown in Fig. 5A–D, the 4 μg dose was well tolerated, with no significant changes in major organs (Supplementary Fig. S7). The splenic changes (white pulp expansion, blurring of the red-white pulp boundary, Fig. 5E ∼ H) are not degenerative lesions but rather reversible pharmacodynamic effects (Supplementary Figs. S7 and S8) of IFN-α-mediated lymphocyte activation, consistent with literature^21^^,^^22^ and with clinical reports showing that interferon-induced splenomegaly resolves after treatment discontinuation.^23^ Notably, the empty LNP control group showed no significant elevation of IL-6 or TNF-α. Therefore, the transient elevation of the cytokines at 3-6 h, returning to near baseline by 17 h (Supplementary Fig. S5), can be attributed to the expression of the exogenous mRNA cargo. This acute-but-self-limited inflammatory response reflects immune activation of the mRNA-LNP platform.^24^ Collectively, the 4 μg dose is well tolerated, with only reversible pharmacodynamic effects and no persistent inflammation.

A single intravenous dose of 4 μg IFN-α14 LNP showed comparable inhibition of HBV antigens to the clinically approved drug PEG-IFNα2 (2 μg, s.c.), with a more sustained suppression of HBsAg at day 7 (Fig. 4B). The two regimens induced similar white pulp expansion in the spleen (Fig. 5E ∼ H, Supplementary Figs. S6 and S7) and comparable serum IFNα concentrations over the measured time course (Fig. 4D). However, the two agents were administered via different routes that strongly influence absorption, distribution, and overall exposure. Moreover, key pharmacokinetic parameters (Cmax, Tmax, AUC, half-life) were not determined for either treatment. Therefore, we cannot conclude that IFN-α14 LNP is superior to PEG-IFNα2. Our conclusion is limited to a regimen-level comparison under the fixed conditions tested (dose, route, and formulation). More comprehensive pharmacokinetic and pharmacodynamic studies of IFN α14 LNP are warranted as future work.

Besides, the lack of functional validation precludes the definitive mechanism by which IFN-α14 LNP exerts its anti-HBV effect. Future studies with more complete controls are needed, including direct comparison of IFN-α14 LNP with IFN-α2 LNP to assess the subtype effect, as well as comparison of the IFN-α14-ApoAI fusion with IFN-α14 alone to evaluate the contribution of ApoAI fusion at the mRNA-LNP level.

We acknowledge that the pilot AAV experiment (Fig. 1C and D) and the dose-finding study (Fig. 3) were performed with limited sample sizes (n = 1-3 per group). Therefore, these data should be interpreted as exploratory and providing a basis for the subsequent studies. The main conclusions of the study are supported by the larger-sample comparative efficacy study (Fig. 4B-C, n = 5 per group) and the comprehensive safety assessment (Fig. 5 and Supplementary Fig. S5–S7; n = 3 per group).

Further optimization of IFN-α14 LNP could prolong expression and enhance efficacy. Recent advances in mRNA engineering offer promising avenues. For instance, the deep generative AI platform GEMORNA^25^ can design mRNA sequences with enhanced translation and stability, achieving up to 41-fold higher reporter expression and 15-fold higher therapeutic protein production. PASylation (fusing a disordered Pro-Ala-Ser polypeptide to protein) has been shown to extend the plasma half-life of IFN-α from ∼44 min to 16 h and to induce functional cure in a murine HBV model.^26^ Applying these technologies to IFN-α14 LNP could synergistically enhance the pharmacokinetics and efficacy, potentially achieving far superior antiviral effects.

Immune activation is a key concern for repeated-dose protein replacement therapies.^27^ In our mouse studies, repeated dosing of IFN-α14 LNP induced neutralizing antibodies (Supplementary Fig. S9), likely due to the xenogeneic nature of human IFN-α14 in mice. In humans, IFN-α14 is a self-protein and should thus benefit from immune tolerance, reducing the risk of anti-drug antibody formation. Importantly, the primary challenge for repeated LNP efficacy is PEG-related immunogenicity—not responses to the endogenous protein—and can be mitigated by formulation optimization.^28^^,^^29^ Thus, the well-established principles of immune tolerance strongly support the translational potential of IFN-α14 LNP for chronic HBV therapy.

In summary, This study demonstrates that LNP-formulated IFN-α14-ApoAI mRNA exerts anti-HBV activity in humanized IFNAR mice with a favorable safety profile. Our findings establish the IFN-α14 LNP regimen as a novel candidate for chronic hepatitis B that warrants further optimization.

## CRediT authorship contribution statement

**Qunling Yang:** Project administration, Methodology, Formal analysis, Data curation. **Qiang Li:** Writing – review & editing, Supervision. **Jie Cao:** Data curation. **Conglin Zhao:** Formal analysis. **Mengxin Lu:** Formal analysis. **Shuangshuang Sun:** Software. **Mingsheng Chen:** Conceptualization. **Chong Chen:** Resources. **Yuxian Huang:** Supervision, Funding acquisition, Conceptualization. **Shuai Tao:** Formal analysis, Data curation, Conceptualization. **Liang Chen:** Writing – review & editing, Supervision, Resources, Project administration, Investigation, Funding acquisition, Conceptualization.

## Funding

This study was supported by Shanghai Association for Science and Technology (21S11905600), ShenKang development center of Shanghai (SHDC12020109), Shanghai Municipal Health Commission (2022YQ027) and the National Health Commission of the People's Republic of China (2026ZD01912301).

## Declaration of generative AI and AI-assisted technologies in the manuscript preparation process

During the preparation of this work the authors used DeepSeek in order to improve language readability and correct grammatical errors. After using this tool, the authors reviewed and edited the content as needed and take full responsibility for the content of the published article.

## Declaration of competing interest

All authors confirm that they have no financial or personal relationships that could influence the work reported in this manuscript.

## Data Availability

Data will be made available on request. edm:link":"https://doi.org/10.6084/m9.figshare.32278026
