# Supplementary material for: Antiviral efficacy of LNP-delivered IFN-α14-ApoAI mRNA for chronic hepatitis B
Source: J Virus Erad. 2026 Jun 3;12(3):100627. doi: 10.1016/j.jve.2026.100627 (PMC13272520; doi:10.1016/j.jve.2026.100627)
Supplement: Multimedia component 1 [file mmc1.docx]

**Supplementary Methods**

|  |
| --- |

**RNA extraction** **Quantitative PCR analysis**

RNA extraction and quantitative PCR (qPCR) analysis were performed as previously described by Fei et al^[1]^. The primers used for detecting HBV 3.5 kb RNA were HBV2268F (5'-GAGTGTGGATTCGCACTCC-3') and HBV2372R (5'-GAGGCGAGGGAGTTCTTCT-3'). For total HBV RNA, the primers were HBV1803F (5'-TCACCAGCACCATGCAAC-3') and HBV1872R (5'-AAGCCACCCAAGGCACAG-3').

**Analysis of HBV plasmid DNA in mouse tissue.**

To extract total nuclear DNA, liver tissues from HDI mice were lysed, and total nuclear DNA was isolated using the Fastpure cell/tissue DNA isolation mini kit (Catalog No. DC102-01, Vazyme, Nanjing, China). pUC18-BPS plasmids were quantified by real-time PCR with the following primers: forward, 5'-GCGGATAACAATTTCACACAGGA-3' (M13R); reverse, 5'-GGGGTTGCGTCAGCAAACACTTGG-3' (HBV 1176–1199). The amplified region spans the pUC18 vector and the BPS HBV sequence.^[2]^ Quantification was performed using the TB Green® premix Ex Taq™ II (Tli RNaseH Plus) kit (Catalog No. RR820, Takara, Japan).

**Hemogram**

Blood samples were collected in Microvette 500 EDTA-coated tubes (BD Biosciences, Franklin Lakes, NJ, USA). Hematological parameters were measured using a Sysmex XN-series Hematology Analyzer (Sysmex Corporation, Kobe, Japan).

**RNA sequencing and data analysis**

Total RNA was extracted from liver tissues using TRIzol reagent (Catalog No.15596026, Invitrogen, USA). Sequencing libraries were prepared using VAHTS mRNA-seq V3 Library Prep Kit for Illumina (Catalog No. NR611, Vazyme, Nanjing, China) following the manufacturer's protocol. The prepared libraries were sequenced on an Illumina NovaSeq 6000 platform by GENEWIZ Inc. (Suzhou, China).

Raw sequencing reads in FASTQ format were processed using Cutadapt (v1.9.1) to remove adapter sequences and low-quality bases (quality score < 20). Clean reads were aligned to the reference genome mm10/GRCm38 using Hisat2 (v2.0.1). Aligned reads were sorted and filtered with SAMtools (v1.8). Gene expression levels were quantified and normalized. Differential expression analysis between groups was performed using the DESeq2 package (v1.34.0) in R, with genes exhibiting |log_2_(fold change)| ≥ 1 and an adjusted P-value ≤ 0.05 considered significantly differentially expressed. Gene Ontology (GO) enrichment analysis of biological processes was conducted using the ClusterProfiler package (v4.2.2). Venn diagrams were generated using the VennDiagram package (v1.7.3) to visualize overlapping differentially expressed genes.

**Selection of interferon‑stimulated genes (ISGs) for heatmap visualization**

To focus on genes central to the interferon response, we compiled a collection of mouse ISGs based on literature mining, the Interferome database (v2.0), and hallmark interferon response gene sets from the Molecular Signatures Database (MSigDB, C7 immunologic signature collection).

From the set of significantly differentially expressed genes between IFN-α14 LNP and PEG-IFN-α2 group, we retained those present in the ISG collection and 27 significantly differentially ISG genes were selected.

The heatmap was generated using the pheatmap R package. Expression values were row‑wise Z‑scored from log₂(FPKM+1) normalized counts. Columns were grouped by treatment, and genes were ordered by log₂(fold change) from up to down‑regulation. All statistical analyses and visualizations were performed in R (v4.1.0).

**Liver and kidney function assessment**

Serum alanine aminotransferase (ALT), aspartate aminotransferase (AST) and creatinine (Cr) levels were measured using commercial kits according to the manufacturer’s instructions. ALT and AST were determined with the ALT/GPT Assay Kit (microplate method, cat. no. C009-2-1) and AST/GOT Assay Kit (microplate method, cat. no. C010-2-1), respectively, from Nanjing Jiancheng Bioengineering Institute (Nanjing, China). Serum creatinine levels were quantified with the Creatinine (Cr) Colorimetric Assay Kit (sarcosine oxidase method, cat. no. E-BC-K188-M; Elabscience, China).

**Cytokine measurement**

Concentrations of interleukin‑6 (IL-6) and tumor necrosis factor‑α (TNF-α) in mouse serum were measured using the Mouse IL‑6 MPCLIA Kit (cat. no. GLM0004) and Mouse TNF‑α MPCLIA Kit (cat. no. GLM0005) from Servicebio (Wuhan, China). All procedures were performed strictly following the manufacturer’s protocols.

**Histopathological analysis**

For histopathological examination, major organs (heart, liver, spleen, lung, and kidney) were collected immediately after sacrifice, fixed in 4% paraformaldehyde, embedded in paraffin, sectioned at 4 μm thickness, and stained with hematoxylin and eosin (H&E). The histology processing was performed by Servicebio (Wuhan, China) and stained sections were digitized using a whole‑slide imaging system (Servicebio, Wuhan, China) and images were captured at the indicated magnifications.

**Detection of serum anti-IFN-α14 antibodies by ELISA**

Briefly, 96-well plates (Catalog No.3590, Coring, NY, USA) were coated with 100 µL/well of recombinant human IFN-α14 (2 µg/mL; catalog No. EHH10371M, Abmart, China) in carbonate buffer (pH 9.6) overnight at 4°C. After blocking with 3% BSA in PBST for 2 h at room temperature, serially diluted serum samples were added and incubated for 2 h at room temperature. Following washing, HRP-conjugated anti-mouse IgG antibody was added and incubated for 1 h. The reaction was developed with TMB substrate, stopped with 2 M H₂SO₄, and absorbance was read at 450 nm.

**Reference**

1. Fei L, Sun S, Yang Q, Huang Y, Li Q, Tao S, et al. **CRISPR/Cas9 System with Dual gRNAs Synergically Inhibit Hepatitis B Virus Replication**. *Discov Med* 2024; 36(185):1169-1179.

2. Shen Z, Yang H, Yang S, Wang W, Cui X, Zhou X, et al. **Hepatitis B virus persistence in mice reveals IL-21 and IL-33 as regulators of viral clearance**. *Nat Commun* 2017; 8(1):2119.

**Supplementary Figures**

**Supplementary Figure S1. HBsAg levels after AAV-IFN-α14 injection.** Serum HBsAg was measured at week 1 before death of AAV-IFN-α14 group (n=3).

**Supplementary Figure S2.** **Quantification of BPS plasmids in mouse liver tissues by qPCR.** Data are from the same mouse shown in Figure 1D. The bar represents the mean of three technical replicates from a single mouse (n=1).

**Supplementary Figure S3. Individual serum HBsAg kinetics of each mouse in the dose‑finding experiment (corresponding to Fig. 3C).** Mice were treated with a single intravenous injection of IFN‑α14 LNP at the indicated doses (0.8, 4, or 20 μg) or control LNPs (EGFP LNP, 20 μg; empty LNP, lipid content matched to 4 μg IFN‑α14 LNP). Each line represents one mouse, labeled as “‑#1” (relatively higher baseline HBsAg) or “‑#2” (relatively lower baseline HBsAg). For the 20 μg dose, one mouse died on day 7 and symbol “X” indicates death. Data were normalized to each mouse’s own baseline (Day 0 = 100%). The lower‑baseline mice showed an earlier decline at day 1 compared to the higher‑baseline mice. No statistical comparisons were performed (exploratory dose‑finding study).


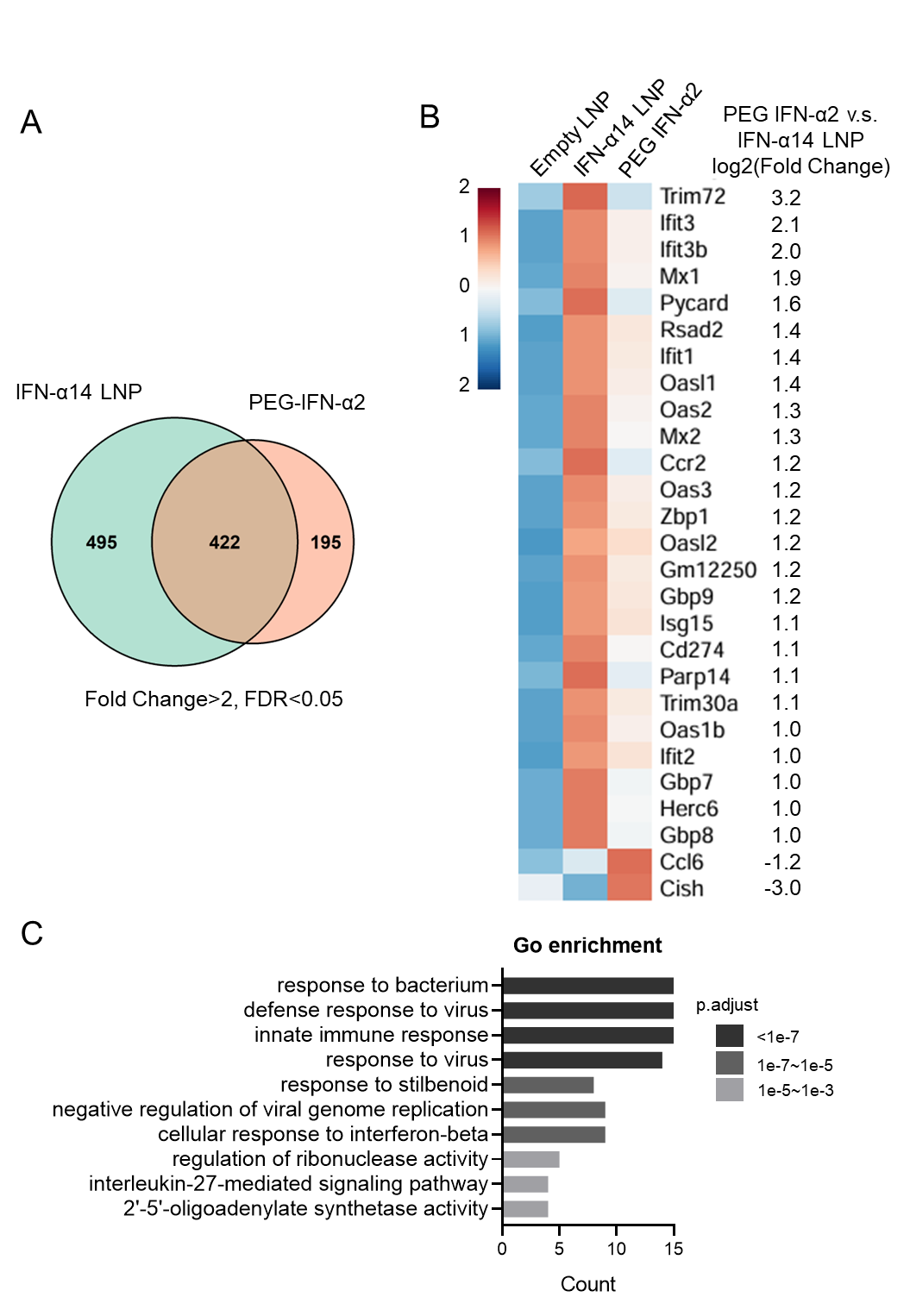


**Supplementary Figure S4. Comparative transcriptomic analysis after treatment with IFN-α14 LNP (4 μg, i.v.) and PEG-IFN-α2 (2 μg, s.c.).** Liver samples were collected at day 2 after a single intravenous injection of empty LNP, 4 μg IFN‑α14 LNP or 2 μg PEG‑IFNα2 (subcutaneously) and RNA‑seq was performed on bulk liver tissue. Lipid content of empty LNP matched to 4 μg IFN‑α14 LNP. (A) Venn diagram showing the number of differentially expressed genes (DEGs) in the livers of mice treated with IFN-α14 LNP or PEG-IFN-α2. (B) Heatmap depicting the expression levels of differentially expressed ISGs between the IFN-α14 LNP and PEG-IFN-α2 groups. (C) Selected GO terms enriched in DEGs between the two treatment groups.


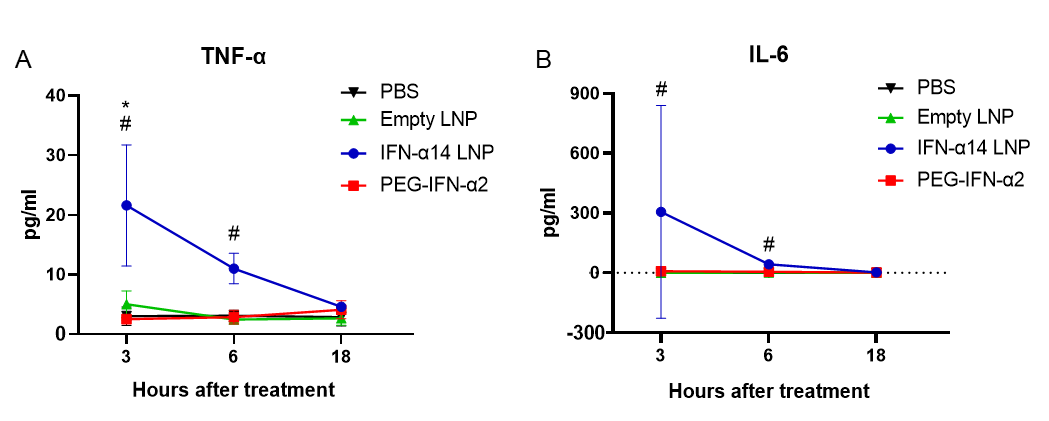


**Supplementary Figure** **S5. Serum IL-6 and TNF-α levels at 3, 6, and 17 hours post-treatment** **(corresponding to Figure 5).** Data are shown as mean ± SD (n=3 per group). Statistical significance was assessed using the Kruskal‑Wallis test followed by Dunn’s multiple comparisons test. *P < 0.05 (IFN-α14 LNP vs. PEG-IFNα2), #P < 0.05 (IFN-α14 LNP vs. PBS or empty LNP).


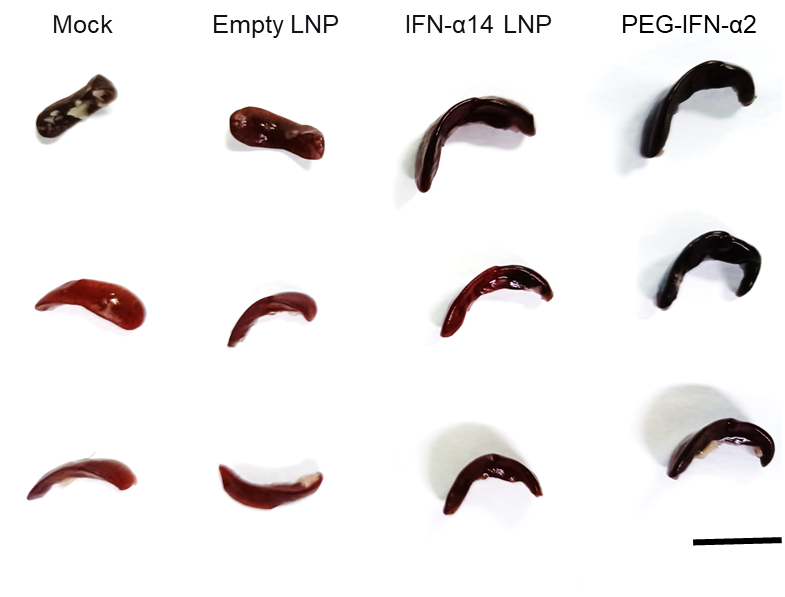


**Supplementary Figure S6. Gross spleen morphology** **(corresponding to Figure 5)****.** Photographs of spleens from each treatment group (n=3 per group). Scale bar = 1 cm (estimated). Spleens from IFN-α14 LNP and PEG-IFNα2 groups were visibly enlarged and darkened.


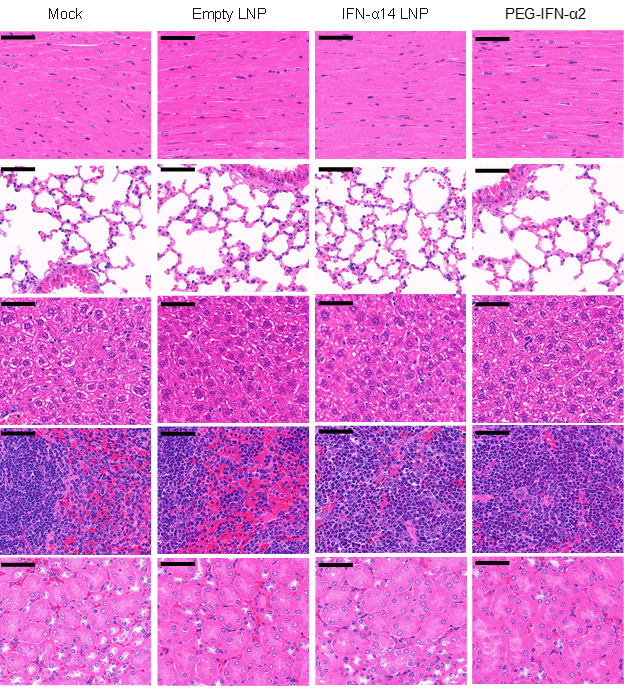


**Supplementary Figure S7. Comprehensive histopathological assessment of major organs** **(corresponding to Figure 5).** Representative H&E-stained sections of heart, lung, liver, spleen, and kidney (from top to bottom) from each treatment group (n=3 per group). Organs were collected on day 9 post-treatment. Original magnification: 40 ×; scale bar = 100 μm. Compared to the control group, no obvious necrosis, inflammation, or structural abnormalities were observed in these organs in either the IFN-α14 LNP or PEG-IFNα2 treatment groups.


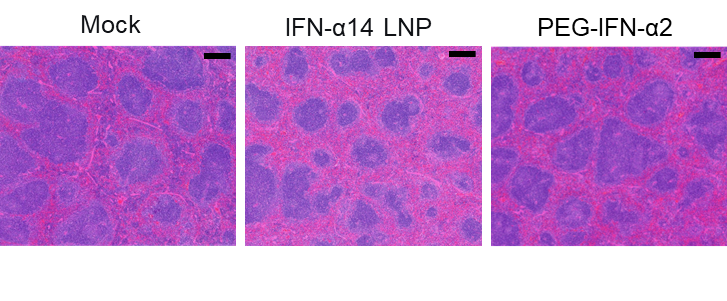


**Supplementary Figure S8. Long-term histopathological assessment of spleen after IFN-α14 LNP treatment (corresponding to Figure 5).** Representative H&E-stained sections of spleens from mice (n=2 per group) four months after a single dose of PBS (mock), IFN-α14 LNP (4 μg, i.v.), or PEG-IFNα2 (2 μg, s.c.). Original magnification: 40 ×; scale bar=250 μm.

**
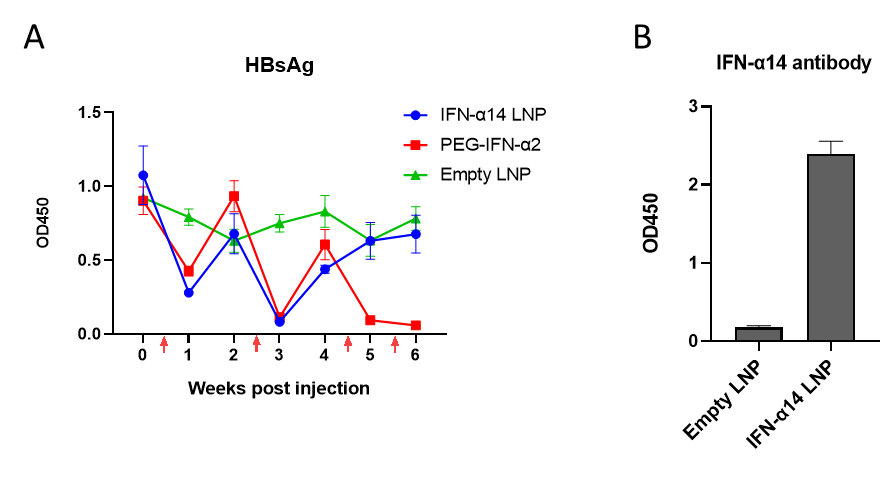
**

**Supplementary Figure S9. Serum HBsAg dynamics and quantification of IFN-α14 antibodies following continuous administration of IFN-α14 LNP.** (A) Serum HBsAg levels over the course of four IFN-α14 LNP treatments; arrows indicate the time points of administration (n=2 per group). (B) Quantification of IFN-α14 antibodies in mouse serum after three administrations of IFN-α14 LNP (n=2 per group).
